# Biochemical Changes in Cardiopulmonary Bypass in Cardiac Surgery: New Insights

**DOI:** 10.3390/jpm13101506

**Published:** 2023-10-18

**Authors:** Luan Oliveira Ferreira, Victoria Winkler Vasconcelos, Janielle de Sousa Lima, Jaime Rodrigues Vieira Neto, Giovana Escribano da Costa, Jordana de Castro Esteves, Sallatiel Cabral de Sousa, Jonathan Almeida Moura, Felipe Ruda Silva Santos, João Monteiro Leitão Filho, Matheus Ramos Protásio, Pollyana Sousa Araújo, Cláudio José da Silva Lemos, Karina Dias Resende, Dielly Catrina Favacho Lopes

**Affiliations:** 1 Residency Program in Anesthesiology, João de Barros Barreto University Hospital, Federal University of Pará, Belém 66073-000, Brazil; victoria.winkler16@gmail.com (V.W.V.); limadobairro@gmail.com (J.d.S.L.); jaime9rodrigues@gmail.com (J.R.V.N.); giovanaescribano@gmail.com (G.E.d.C.); jordanadecastro@hotmail.com (J.d.C.E.); sallatielcabral@icloud.com (S.C.d.S.); jonathan2almeida@hotmail.com (J.A.M.); felipe.rud@gmail.com (F.R.S.S.); jfilho_monteiro@icloud.com (J.M.L.F.); karinaresendeanestesio@gmail.com (K.D.R.); 2 Laboratory of Experimental Neuropathology, João de Barros Barreto University Hospital, Federal University of Pará, Belém 66073-000, Brazil; 3 Hospital Israelita Albert Einstein—HIAE, São Paulo 05652-900, Brazil; matheus.ramos07@hotmail.com; 4 Department of Cardiovascular Anesthesiology, Hospital Clínicas Gaspar Vianna, Belém 66083-106, Brazil; pollyanestesia@gmail.com (P.S.A.); claudiosau@gmail.com (C.J.d.S.L.)

**Keywords:** cardiopulmonary bypass, inflammation, lactate, cytokines, cardiac surgery

## Abstract

Patients undergoing coronary revascularization with extracorporeal circulation or cardiopulmonary bypass (CPB) may develop several biochemical changes in the microcirculation that lead to a systemic inflammatory response. Surgical incision, post-CPB reperfusion injury and blood contact with non-endothelial membranes can activate inflammatory signaling pathways that lead to the production and activation of inflammatory cells, with cytokine production and oxidative stress. This inflammatory storm can cause damage to vital organs, especially the heart, and thus lead to complications in the postoperative period. In addition to the organic pathophysiology during and after the period of exposure to extracorporeal circulation, this review addresses new perspectives for intraoperative treatment and management that may lead to a reduction in this inflammatory storm and thereby improve the prognosis and possibly reduce the mortality of these patients.

## 1. Introduction

Cardiopulmonary bypass was used for the first time in cardiac surgery over 60 years ago, and since then, many advances have occurred in the conduct of CPB and cardiac anesthesia [1]. CPB comprises a set of devices and techniques that replace cardiac and pulmonary function during surgery. Several cardiopulmonary bypass methods are used for mechanical support of respiratory and cardiocirculatory failure, so that there is adequate blood flow to supply oxygen to the vital organs [2]. CPB makes it easier for procedures that need to enter the intracardiac space, such as valve replacement, to be performed with a reduced amount of blood and under controlled conditions [3].

Substantial evidence from several studies on CPB reports that this procedure stimulates the inflammatory process and that generates reactive oxygen and nitrogen species that overcome endogenous antioxidants, resulting in increased oxidative stress that significantly affects the rates of mortality and postoperative morbidity [1,3,4,5,6,7,8]. Several factors seem to be the cause of the systemic inflammatory reaction, such as blood contact with the CPB device’s surface, surgical trauma, endotoxemia, blood loss and ischemic reperfusion injury [9]. Thus, there is activation of the complement system and the immune system, leukocytes and endothelial cells, which, in turn, are responsible for the release of multiple pro-inflammatory cytokines [10]. Some deleterious effects that influence the high mortality rate are related to the inflammatory response syndrome which, to a large extent, is related to the interface of blood components, air and the artificial surfaces of the device [2,11]. Despite significant advances in recent years, oxidative stress and inflammation remain major concerns when using CPB [12].

Cardiac surgery with cardiopulmonary bypass is associated with systemic inflammatory response, a clinical condition that is characterized in some cases with severe hypotension due to low systemic vascular resistance during and after cardiopulmonary bypass, in which some of these cases do not respond to volume or catecholamines. This condition is known as vasoplegic syndrome [11,13]. The pathophysiology is complex and includes, in addition to the intense inflammatory response, dysregulation of the vasodilator and vasoconstrictor properties of vascular smooth muscle cells [11]. Although norepinephrine is confirmed as a first-line therapy for the treatment of vasoplegia, currently, many randomized studies have identified new adjuvant therapies to control metabolic and oxidative stress as a pharmacological strategy to reduce the incidence of vasoplegic syndrome [11].

In this narrative review, we will present our study, focusing on the inflammatory response, vasoplegic syndrome and new insights regarding adjuvant and non-catecholaminergic treatment for the inflammatory response generated during the cardiopulmonary bypass method.

## 2. Search Strategy

We used PubMed, Web of Science and Embase to explore/find studies pertaining to cardiopulmonary bypass. The search was implemented by using the following keywords: “cardiopulmonary bypass”, “oxidative stress”, “inflammation”, “coagulation”, “ischemia/reperfusion”, “vasoplegic syndrome”, “antioxidants”, “free radical”, “cardiac surgery”, “lung/kidney injury”. In that context, we conducted a literature search in the PubMed search engine (https://pubmed.ncbi.nlm.nih.gov (accessed on 27 June 2023)). This narrative review is the result of the works having been thoroughly scrutinized by specialists in the field, in order to critically include or exclude them.

In this review, we included all studies which involved cardiopulmonary bypass. We did not restrict the types of articles, and we included peer-reviewed studies, book chapters, reviews, letters to editors and animal studies. The studies included in this review were all limited to English only. We excluded studies that did not focus on cardiopulmonary bypass and non-English studies.

## 3. Pathophysiology of a Cardiopulmonary Bypass

During a CPB, the pumping functions of the heart are performed by a mechanical pump and the functions of the lungs are replaced by a device capable of performing gas exchange with the blood. In this context, to understand the systemic complications related to this procedure, it is necessary to understand the blood circuit during cardiopulmonary bypass [14].

In CPB, venous blood is diverted from the heart and lungs, arriving at the right atrium of the patient through cannulas inserted in the superior and inferior vena cava. Through a single channel, venous blood is taken to the oxygenator, a reservoir made of semipermeable membranes to separate blood from oxygen and perform gas exchanges [15,16].

From the oxygenator, blood is directed to a part of the patient’s arterial system, usually the ascending aorta, where it travels through the arterial system and is distributed to all organs, supplying oxygen to the tissues for carrying out vital processes and removing the carbon dioxide produced by them. After circulating through the tissue capillaries, the blood returns to the superior and inferior vena cava system, where it is continuously redirected to the CPB machine until the end of the surgery [16].

CPB control is carried out by means of a machine, which aspirates and propels the blood. The machine consists of a control panel, oxygenator, reservoir, arterial pump that replaces the contractile function of the heart, cardioplegia (system for mixing blood and cardioplegic solution) and tubes or cannulas (arterial and venous) [15].

**Figure 1:**
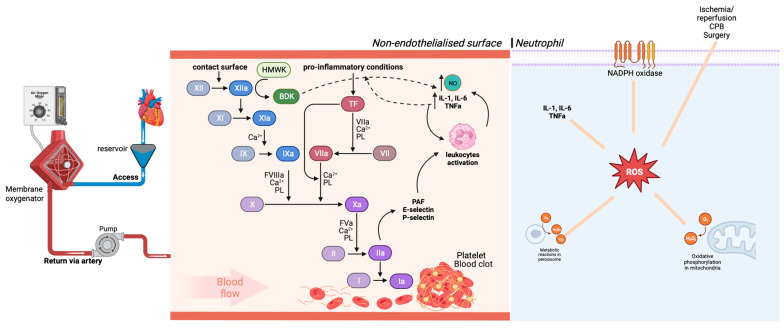
**Activation of the coagulation pathway and oxidative stress in cardiopulmonary bypass** (**CPB**)**.** The contact surface is responsible for producing activated factor XII (FXIIa), which induces the intrinsic coagulation pathway, leading to thrombin formation. Factor XIIa converts the high-molecular-weight kininogen (HMWK) into bradykinin. Bradykinin stimulates the release of nitric oxide and inflammatory cytokines. Cytokines stimulate the extrinsic pathway of coagulation and potentiate the formation of thrombin and clot formation and have direct effects on leukocytes. CPB initiates multiple processes that stimulate the production of reactive oxygen species (ROS). The main forms of cardiac ROS are superoxide (O_2_^−^) and hydrogen peroxide (H_2_O_2_).

After opening the patient’s chest, the surgeon introduces the cannulas into the right atrium and the inferior and superior vena cava. Thus, oxygen-poor blood is diverted and directed to a reservoir (Figure 1). In the machine, the heat exchanger rewarms or cools the blood as needed. Then, an oxygenator removes carbon dioxide from the blood and adds oxygen. After that, the blood passes through a filter that removes air bubbles and other emboli before returning to the body through a pump that directs the blood to the aorta. [17]. The role of arterial cannulation, performed by inserting a cannula into an artery, is to return blood to the patient’s circulation. Before returning to the body, the blood is filtered to ensure that no particles, debris, or gaseous emboli enter the circulation [15,16].

The function of the arterial pump is to replace the function of the heart. It sends the blood from the reservoir and ensures an artificial blood circulation. The way in which blood flow should be provided, continuous or pulsatile, has been the subject of debate, which continues to this day [15].

### 3.1. Systemic Response to CPB

In cardiac physiology, the cardiovascular system is a complex set of vessels, which driven by the heart, make the blood circulate throughout the body. The veins are responsible for the flow in centripetal way, whereas the arteries are responsible for the flow in centrifugal way. Within this system, the microcirculation—venules, arterioles and capillaries—is the place where gas exchange occurs and where the regulatory mechanisms of peripheral blood flow are located [18].

During CPB, the physiology of the circulation is completely modified by the introduction of a non-pulsatile flow on the arterial side, which opposes an elevated venous pressure on the venous side of the circulation. This situation generates adaptation mechanisms, thus providing a “shunting” effect, sometimes harmful to the circulation, which may result in the development of systemic inflammatory response syndrome (SIRS) [18].

It is believed that factors associated with CPB, such as hemodilution, contact activation and induction of systemic inflammatory response, impair microcirculatory perfusion by affecting both transport and diffusion of oxygen at the microvascular level [19].

Another form of damage to the microcirculation related to the use of CPB is the formation of microbubbles, which circulate in the bloodstream and lodge in the capillaries, causing obstruction, promoting ischemia, inflammation, complement activation, platelet aggregation and clot formation [20]. In addition, CPB is responsible for other changes in circulation, such as replacement of pulsatile physiological flow to continuous, which increases the pressure on the venous side. In the microcirculation, the continuous flow induces to phenotypic cell adaptation that also may result in the development of SIRS [18].

Hypothermia associated with cardiopulmonary bypass aims to reduce the metabolic needs of patients, offering additional protection to the body, especially the vital organs, avoiding anoxia injuries [21]. However, hypothermia reversibly inhibits clotting factors and platelets, and rapid rewarming and hyperthermia are associated with brain injury [22].

In the first moments of CPB, hypotension is common due to the reduction in the perfusion flow, the reduction in blood viscosity due to hemodilution, and the increase in bradykinin. After this period, the body begins a compensatory response that, particularly with hypothermia, the elevation of systemic vascular resistance and the absence of pulsatility in the circulation, result in hypertension [23].

However, consequently, renal vasoconstriction occurs, predisposing the kidneys to ischemia and injury [24]. In addition, hemodilution with crystalloid solutions, when in excess, predisposes the patient to the formation of edema and watery diuresis rich in electrolytes, favoring hydroelectrolytic imbalance [25,26].

Hemorrhagic disorders related to CPB are related to changes in blood clotting, since blood circulates through tubes and devices that are non-endothelial surfaces. This imbalance of blood hemostasis during CPB is the most common occurrence of thrombotic events, while, after CPB, bleeding is usually reported [23,26,27].

Regarding the lungs, there is an increase in water leakage into the interstitium due to inflammatory cells, surfactant inactivation and atelectasis and reduction in lung capacity, which, together with exposure to hypothermia maintained during CPB, cause damage to the pulmonary endothelium [28,29].

### 3.2. Metabolic Response

Patients undergoing CPB are subject to a significant hydroelectrolytic imbalance. The migration of water between the different compartments depends on the concentration of electrolytes for that the body’s water balance is maintained. Thus, when the patient undergoes CPB, important imbalances may occur, such as excessive hydration, due to the increased number of crystalloids in the perfusate [30].

The hyperhydrated patient may present facial or generalized edema, ascites, pleural effusion, respiratory failure, asthenia, disorientation, delirium and seizures or other neurological manifestations. Hyperhydration is a complication that is accentuated in patients with low amounts of proteins in the body, representing another risk factor for this individual when performing surgical procedures, considering that the oncotic pressure of the plasma is reduced and allows extravasation of liquids from the plasma to the interstitial space, especially if the liquid supply is not adequately dimensioned [31,32].

In this context, sodium (Na^+^) is the main ion for sustaining water balance, since its loss can cause a reduction in extracellular osmotic pressure and, consequently, cause the transition from this compartment to the intercellular compartment. However, if there is an increase in extracellular sodium levels, there is an increase in osmotic pressure and, consequently, this results in the interstitial accumulation of water, with the development of edema [31,32].

Another intracellular electrolyte is potassium (K^+^), which is responsible for conducting the electrical impulse and performing muscle contraction. Its unbalanced extracellular accumulation, characterized by hyperkalemia, and can be harmful, reducing electrical conduction and myocardial contraction strength, that is, causing cardiac arrest, which demonstrates its significance in cardiorespiratory surgical procedures in patients undergoing CPB, especially during the infusion of the cardioplegia solution [31,32].

Other studies are in the same direction, highlighting calcium (Ca^2+^), as a fundamental electrolytic substance for bone formation and blood flow regulation, meaning that its lack characterizes hypocalcemia and can cause the same risks mentioned in relation to hyperkalemia, that is, it can cause a cardiac arrest, resulting in death, and the balance of the amount of calcium also tends to reduce the risk of blood clotting during and after the surgery [31,32]. 

Magnesium (Mg^2+^) is an important electrolyte in activating metabolism, including glycemic and protein metabolism, in addition to enabling neuromuscular contraction, but, in high concentration, hypermagnesemia causes risks, with regard to unbalanced muscle relaxation, such as in the heart muscles, as well as causing cardiac disorders related to the process of electrical conduction [33].

Hydroelectrolytic alterations may therefore mean imminent risks to surgically assisted cardiac patients, so that when undergoing CPB, the individual must have balanced systemic functions or, on the contrary, when identifying the respective alterations, the artificial organs must restore the balance [30].

An elevation of lactate levels (hyperlactatemia) is detectable in 10% to 20% of patients undergoing CPB and is associated with adverse effects such as increased morbidity and mortality [34]. Type A hyperlactatemia is the most common type in patients after cardiac surgery and is strongly associated with metabolic acidosis. It results from anaerobic metabolism, when the supply of oxygen is reduced below the requirement of cellular metabolism, resulting in tissue hypoxia [35].

Some factors that lead to dysoxia during CPB and culminate in increased lactate levels are comorbidities intrinsic to the patient, such as preoperative dehydration, high levels of preoperative serum creatinine, active endocarditis, congestive heart failure, low left ventricular ejection fraction, hypertension, atherosclerosis, low preoperative and perioperative hemoglobin values and preoperative low cardiac output [36].

The reference range given for blood lactate is 0.5–2.2 mmol/L under certain physiological conditions, with some variation between authors. Alert levels are usually defined as being above 3 mmol/L during CPB, while other authors point out that a lactate peak above 4.0 mmol/L is a better predictor of morbidity and mortality. And there are still those who suggest that a situation of significant hyperlactatemia has a level greater than 5 mmol/L [37].

Lactate metabolism is closely related to glucose metabolism, as both compounds are used to biosynthesize each other. Therefore, metabolic disorders that affect glucose metabolism alter lactate homeostasis [38]. In cardiac surgeries, poor glycemic control is associated with the induction of type B hyperlactatemia in the perioperative period and studies show that low levels of glucose and high levels of lactate are closely related in these patients and, commonly, levels are spontaneously regulated up to 24 h [39].

### 3.3. Systemic Inflammatory Reaction

CPB is associated with microvascular alterations in several pathological aspects. Endothelial cell injury and consequent acute inflammation with vascular damage, alteration of the coagulation cascade, reperfusion injury and gaseous microemboli corroborate organ dysfunctions during and after CPB [40].

The pathophysiology of the systemic inflammatory response to CPB is multifactorial and can be divided into two main phases: “early” and “late”. The first phase occurs when blood makes contact with non-endothelial surfaces of the system cannulas (“contact activation”). The late phase is caused by ischemia-reperfusion injury (I/R injury), endotoxemia, coagulation disorders and heparin/protamine reactions [41].

### 3.4. Endothelial Injury

The endothelium is a participant in different physiological functions, including the control of vascular tone and permeability, hemostasis and immune system responses. CPB can lead to an inflammatory state similar to SIRS, with endothelial damage [42]. 

There are two main mechanisms of endothelial injury—neutrophil-mediated and non-neutrophil-mediated. In the first, integrins on the surfaces of neutrophils bind to molecules on endothelial cells, generating oxidative stress due to the reduction of ferric iron to ferrous iron caused by superoxide anion, which is generated from xanthine oxidase, produced by neutrophil elastase introduced into endothelial cells [40,43]. Neutrophil-mediated endothelial cytotoxicity can also be caused by intracellular mechanisms involving nitric oxide synthase [44].

In non-neutrophil-mediated injury, circulating pro-inflammatory cytokines (TNFa and IL-1) directly stimulate endothelial cells, leading to a pathological increase in permeability, causing tissue edema and impaired oxygen exchange, causing multiorgan dysfunction [45].

### 3.5. Alteration of the Coagulation Cascade

Contact with the artificial surface of the circuit leads to activation of the coagulation cascades (Figure 1) and the alternative complement pathway. Activated factor XII (XIIa) leads to the generation of bradykinin and the activation of the intrinsic coagulation pathway; bradykinin is a potent vasoactive peptide that alters endothelial permeability, smooth muscle tone and induces the production of cytokines and nitric oxide [46]. As artificial surfaces, unlike the endothelium, have no regulatory molecules for suppressing the complement system; they lead to an excessive inflammatory response and capillary leakage, which has been demonstrated as a complication of cardiopulmonary bypass [46].

Furthermore, cytokines generated from brakinin induction may be able to activate the extrinsic pathway of coagulation and leukocytes. Together, there is an increase in the common pathway of coagulation, so activated factor X (FXa) converts prothrombin (II) to thrombin (IIa), which then cleaves fibrinogen (I) to fibrin (Ia), resulting in subsequent formation of clots [47,48]. Factor IIa increases the expression of adhesion molecules, such as platelet activating factor (PAF), P-selectin and E-selectin by endothelial cells, increasing adhesion and activation of defense cells, such as neutrophils [49].

The factor Xa and the thrombin formed, even with the administration of heparin, are related to inflammatory processes and tissue remodeling. Thus, coagulation is closely related to inflammatory processes during CPB [50].

### 3.6. Oxidative Stress

Aortic clamping in CPB blocks coronary flow with a decrease in oxygen supply to myocytes, altering electrical activity and ceasing cardiac mechanical activity. This maneuver of aortic clamping and subsequent removal of the clamp in CPB provides favorable conditions for the formation of free radicals, triggering oxidative stress [12,51].

In ischemia, the supply of oxygen to the mitochondria ceases, interrupting the Krebs cycle. Thus, the generation of ATP becomes primarily anaerobic. This change is accompanied by an increase in cytosolic lactate and a reduction in intracellular pH. The reduction in the cellular concentration of ATP interrupts the activity of active pumps that are important in ionic homeostasis, such as the sodium and potassium pump and the sarcoplasmic reticulum calcium,S ATPase, resulting in cytosolic overload of Na^+^ and Ca^2+^, which prevents cell repolarization, leading to contractile dysfunction. Additionally, high concentrations of Ca^2+^ in the cytosol activate enzymes associated with lipid peroxidation, production of reactive oxygen species (ROS), dysfunction of contractile proteins, loss of cell function and, ultimately, cell death [52,53].

In tissue reperfusion with the end of aortic occlusion, it promotes the formation of ROS in the cytosol, mitochondria, peroxisome, lysosomes and plasmatic membrane of polymorphonuclear leukocytes activated during the CPB ischemia period. Thus, the reintroduction of the oxygen molecule in the ischemic heart tissue causes free radicals to react with polyunsaturated fatty acids from the cell membrane. This starts a chain of oxygen-dependent lipid deterioration where there formation of lipid peroxides and hydroxy-peroxides occurs, which are generated rapidly by the NADPH oxidase complex in response to cytokines that result in the generation of excessive amount of ROS, decreased membrane fluidity and increased permeability and consequent damage to the cell membrane, contributing to functional impairment in CPB [53,54,55].

When these ROS accumulate disproportionately to the body’s antioxidant capacity, we are facing a situation called oxidative stress. The excess of reactive species causes arrhythmias, reduction of the systolic function and change in the permeability of the myocytes membrane. To avoid this, hydroperoxide radicals are removed from cells by enzyme systems with antioxidant functions, generally present in the myocardium. These enzyme systems with antioxidant action are responsible for limiting the intracellular accumulation of reactive species during normal metabolism, reducing oxidative damage to proteins, lipids and DNA [53,54,55,56,57].

## 4. Vasoplegic Syndrome Associate to CPB

One of the main changes that occur in patients undergoing cardiac surgery with cardiopulmonary bypass is the vasoplegic syndrome. It is characterized as a circulatory shock, of the distributive type, coursing with systemic vasodilation. It has a pathophysiology and treatment similar to that of sepsis, with some peculiarities [58,59,60,61]. In this sense, the vasoplegia observed after CPB can affect a considerable number of patients, with studies showing an incidence greater than 50%, and is characterized by a low systemic vascular resistance, with normal or slightly increased cardiac output that occurs in the first 24 h, associated with a cardiac index (CI) greater than 2.2 L/kg/m^2^. This drop in SVR leads to tissue hypoperfusion and progression to organ dysfunction [62,63]. The treatment of this condition is based on the use of vasopressors to maintain mean arterial pressure within recommended values, and recent research has associated the use of new, non-catecholaminergic strategies as adjuvant treatments. 

Furthermore, statistics indicate that approximately 25% of patients undergoing cardiac surgery with cardiopulmonary bypass present vasoplegic syndrome, with variations depending on the associated risk factors, and with high rates of morbidity and mortality in these patients [64,65]. The mechanisms by which CPB leads to vasoplegia are multifactorial and many signaling pathways are still being studied, but factors inherent to patients such as obesity, diabetes, autoimmune diseases, intraoperative hyperthermia seem to contribute to the increased incidence, in addition to the prolonged duration of CPB [64].

### 4.1. Regulatory Mechanisms: Vasoconstriction and Vasodilation

**Figure 2:**
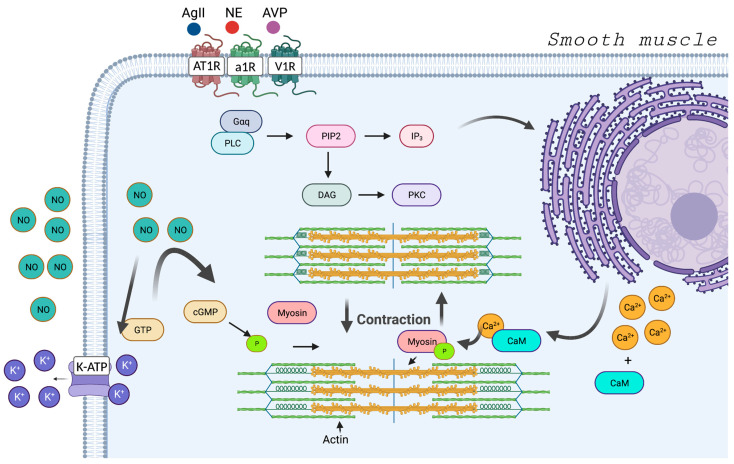
**Physiology of contraction and relaxation of vascular smooth muscle.** Muscle contraction occurs in response to the activation of receptors present in the membrane, such as the alpha-1 adrenergic receptor (a1R), the vasopressin-1 receptor (V1R) and the angiotensin type-1 receptor (AT1R); their activation by the selective agonist promotes the phosphorylation of the Gq protein and with that, the signaling pathway starts. With this, there is the release of calcium by the endoplasmic reticulum through phosphatidylinositol-3 (IP3); the calcium released in the cytosol binds with calmodulin (CaM), forming the calcium-calmodulin complex that phosphorylates myosin and promotes contraction. On the other hand, the nitric oxide (NO) produced by the endothelium reaches the smooth muscle, converting GTP into cGMP. cGMP dephosphorylates myosin and promotes relaxation. In addition, NO activates ATP-sensitive potassium channels (KATP), leading to hyperpolarization and inhibition of vasoconstriction. Angiotensin II (AgII); norepinephrine (NE); arginine-vasopressin (AVP); cyclic guanosine monophosphate (cGMP); guanosine triphosphate (GTP); diacyl glycerol (DAG); phospholipase C (PLC); phosphatidylinositol-4,5-bisphosphate (PIP2); protein kinase C (PKC).

Physiologically, the dynamics of vascular smooth muscle contraction are strongly linked to the calcium ion. It occurs due to an intracellular response to the increase in this positive ion, which forms a complex with calmodulin, and this complex activates signaling pathways that lead to the phosphorylation of the myosin light chain that will bind to actin, promoting the shortening of muscle fibers and the contraction effect (Figure 2). There are several ways in which increased intracytoplasmic calcium can occur; the main ones are through G protein-linked receptors, of the Gq type, in which the activation of this receptor internally signals the cell to mobilize calcium from the endoplasmic reticulum; the responsible receptors are alpha-1 adrenergic receptor, vasopressin-1 receptor and type 1 angiotensin receptor [66,67].

Norepinephrine (NE), which is the endogenous ligand of the alpha-1 adrenergic receptor, is released from nerve endings originating from the sympathetic chain, and epinephrine, a derivative of NE, is released from the adrenal and is also capable of binding to the alpha-1 adrenergic receptor. Arginine vasopressin (AVP) is released from the hypothalamic axis and angiotensin II is regulated as part of the renin–angiotensin–aldosterone axis [66,67]. All these signals are regulated in response to stress and can be pharmacologically modulated.

To cause vasodilation or in the absence of vascular muscle contraction, it is possible to block the receptors or through the production of nitric oxide (NO) [44,68]. It is produced from the enzymatic induction of nitric oxide synthase (NOS), which converts L-arginine into NO. There are three isoforms of NOS, eNOS, present in the endothelium and offer a constant production of NO to endothelial cells that can rapidly diffuse to vascular smooth muscle cells and exert its vasodilation effects [68]. The iNOS, enzyme is inducible via oxidative stress and inflammatory cytokines (interleukin 1, tumor necrosis factor alpha and interferon gamma) and can produce significantly higher levels of NO when compared to eNOS [66,69,70,71]. These high levels of NO can react with superoxide, leading to peroxynitrite formation and cellular toxicity [72]. And, finally, nNOS, present in neuronal cells and participates in functions such as neuronal plasticity and regulation of cerebral perfusion pressure, with mechanisms of vasoconstriction and relaxation of cerebral arteries, but this isoform of NOS participates in few of the systemic mechanisms of vasodilation induced by NO [73].

NO promotes vasodilation through several methods, the main one being related to the activation of guanalyl cyclase, an enzyme found in vascular smooth muscle that catalyzes the dephosphorylation of guanosine triphosphate to cyclic guanosine monophosphate (cGMP), which inhibits calcium entry through voltage-gated channels and activates intracellular proteins that are cGMP-dependent [62]. NO also activates ATP-sensitive potassium channels (KATP), which promote potassium efflux and induces the cell to a hyperpolarized state. In a hyperpolarized state, the secondary intracellular cascade that leads to vasoconstriction is inhibited [74].

### 4.2. Vasoplegia

**Figure 3:**
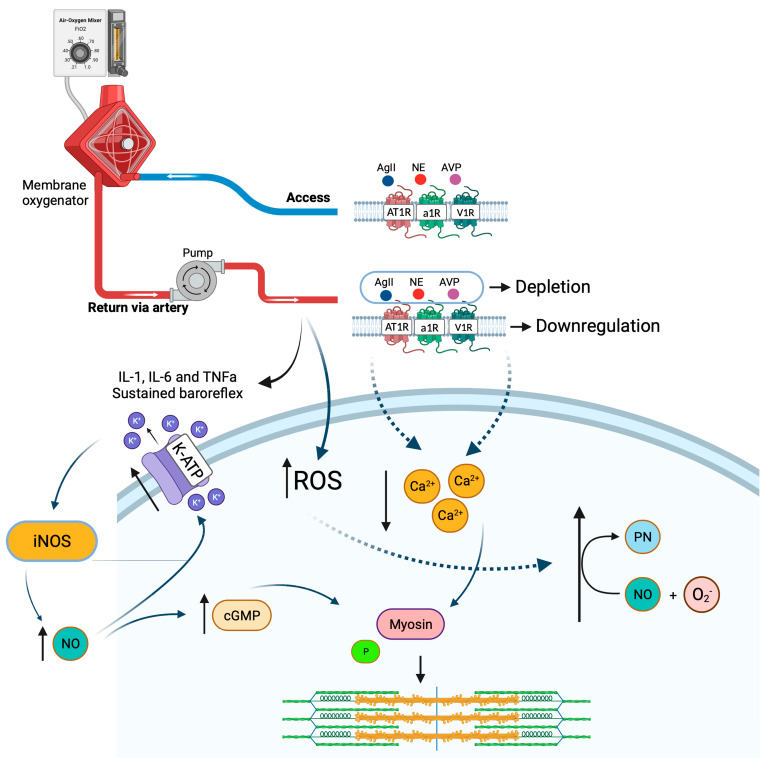
**Pathophysiology of vasoplegia.** With the return of blood to the systemic circulation after passing through the oxygenation membrane during cardiopulmonary bypass, there is a depletion of the endogenous ligands of the alpha-1 adrenergic receptors, vasopressin-1 and the type-1 angiotensin receptor, in addition to the downregulation of the receptors themselves, making vasoconstriction an event that is unlikely to occur. The reduction in receptor concentration leads to a reduction in intracellular calcium and with it, a reduction in contraction. In addition, CPB induces the production of reactive oxygen species (ROS) and releases inflammatory mediators that can lead to the desensitization of adrenoreceptors and induces the production of nitric oxide (NO). NO leads to an increase in cGMP, which inhibits calcium in cells, leading to muscle relaxation. NO also activates ATP-sensitive potassium channels (KATP), leading to hyperpolarization and inhibition of vasoconstriction. NO further reacts with the superoxide anion radical (O_2_^−^) to form peroxynitrite (PN).

These regulatory mechanisms of vasoconstriction are deregulated, to a greater or lesser extent, during and after the cardiopulmonary bypass ends (Figure 3). The passage of blood through CPB stimulates the activation of the complement cascade, production of ROS species and release of inflammatory mediators, such as the triad of cytokines, interleukin-1 (IL-1), interleukin-6 (IL-6) and tumor necrosis factor-alpha (TNF-α) [45,75,76,77,78]. All this inflammatory biochemistry is capable of acting in specific areas of the brain such as the locus coeruleus and the paraventricular nucleus of the hypothalamus, where the cells responsible for the hypothalamic-pituitary-adrenal axis are located; they stimulate these regions and lead to a reduction and desensitization of the alpha-1 adrenergic receptor and an increase in the inflammatory state, thus forming a cycle that is difficult to overcome (11). This reduction in receptors may be related to reduced gene expression in response to inflammation [66] or, more significantly, receptor desensitization, triggered by exaggerated catecholamine release in response to baroreflex-dependent stimulation [11,66].

These inflammatory mediators can also increase the production of nitric oxide (NO) through the induction of iNOS by activating the nuclear factor, kappa B (NF-κB), which is a vasodilator and, in excess, can result in vasoplegic shock [79,80,81]. As a response to shock, the body stimulates the release of vasopressors via another region of the hypothalamus and the renin–angiotensin–aldosterone system in the production of angiotensin II to try to maintain the contraction of vascular smooth muscle and tissue perfusion, but in the persistence of shock, these mechanisms suffer depletion and saturation [82,83]. Despite this, vasopressin is of considerable importance in the process of controlling vasoplegia, as it is capable not only of neutralizing the effects of NO, but also of decreasing NO production [61,84].

Although the body maintains internal control mechanisms in response to vasoplegia, such as the release of vasopressors and sustained activation of the sympathetic system [11], recent studies have shown that circulating levels of vasopressin are reduced a few hours after the cardiopulmonary bypass, suggesting that CPB may deplete this vasopressor and contribute to the installation/maintenance of vasoplegic syndrome [85,86]. Not only vasopressin, but the activity of several types of K^+^ channels promotes potassium efflux and membrane hyperpolarization. Among these channels is the ATP-sensitive K^+^ channel (KATP) [74]. Activation of this channel has been strongly linked to the development of pathological vasodilation [62]. Several mechanisms may explain the activation of the KATP channel in the vasoplegic syndrome associated with CPB, including NO release, vasopressin deficiency, hypoxia and acidosis [62,86,87].

In addition, the surgical incision is initially capable of triggering an inflammatory response, even if to a lesser extent, when compared to CPB. As the surgery progresses and the patient is coupled to the cardiopulmonary bypass system, systemic inflammatory response syndrome can occur [80]. Of the generated cytokines, it is believed that interleukin-6 is the one most associated with vasoplegia, as it is notably a potent inhibitor of vascular contraction [64,88,89]. As the contact time of the blood with the CPB equipment is one of the factors that contribute to the degree of the inflammatory response generated, as it continues, the secondary immune response occurs as a result of the reinfusion of blood from the circuit to the aorta. A cluster of hemolyzed cells and injured platelets stimulate a secondary immune response leading to increased inflammation and subsequent loss of vascular tone [80,90].

In refractory cases of vasoplegic syndrome, an increase in the dose of vasopressors is necessary; however, without effect on mean arterial pressure levels, the patient can return to CPB and a new attempt to withdraw from CPB can be performed. This may occur, as the inflammatory state generated may be capable of inhibiting the response of adrenergic receptors to catecholamines, through mechanisms that are still poorly understood [66].

## 5. Future Perspective and Antioxidant Agents

Although there are endogenous control systems to reduce the oxidative stress generated by cardiopulmonary bypass, they alone are often unable to attenuate the damage caused [91]. There are considerable biochemical changes during and after the CPB, such as oxygenation in non-endothelial membranes, reperfusion damage, absorption of nutrients in the CPB circuit [91,92]. This context of inflammation and oxidative stress has been associated with postoperative complications in these patients, such as atrial fibrillation, acute kidney and lung and liver injury [12,51,93,94,95]. In this sense, several drugs with antioxidant properties are being investigated, either as individual therapies or in combined treatment to reduced oxidative damage.

### 5.1. Miniaturized Cardiopulmonary Bypass

The miniaturized cardiopulmonary bypass (mCPB) method was developed as a more biocompatible alternative to conventional cardiopulmonary bypass [96]. It consists of a small, closed, heparin-coated circuit in which venous blood is returned to a diffusion membrane oxygenator through active drainage, which reduces mechanical trauma. Although some studies have shown considerable benefit in relation to conventional systems, mainly in the reduction of the inflammatory response and its associated complications [97,98], meta-analyses of randomized clinical studies show contrasting results, in which there is no significant difference in the incidence of primary outcomes such as stroke and mortality among mCPB compared with the CPB [99].

### 5.2. Low-Level Light Therapy

As already mentioned, oxidative stress triggers several events that can lead to negative outcomes in the patient [12]. Although new methods have been developed, such as mCPB and materials with better biocompatibility, the results are still uncertain. In this regard, although premature, the use of low-level light therapy (LLLT) in the red-to-near-infrared radiation has shown promise [100,101,102,103]. The results of this method include a reduction in the inflammatory response, as lipid peroxidation, and hemolysis during cardiopulmonary bypass [100]. Furthermore, it has been shown to be important in also reducing platelet loss and change of pattern of aggregation and CD62P (P-selectin) expression. This suggests that LLLT can stabilize platelet function during CPB and decrease the side effects associated with the interaction of cells with an non-endothelial surfaces [101].

### 5.3. Dexmedetomidine

Some anesthetics may extend clinical benefits beyond anesthesia and may also offer anti-inflammatory support [104]. Dexmedetomidine (DEX), an alpha 2-adrenergic receptor agonist, has been studied as a possible modulator of the inflammatory response caused by cardiopulmonary bypass in cardiac surgery [105,106,107]. Bulow et al. [107] demonstrated that the use of dexmedetomidine attenuated the increase in inflammatory cytokines (IL-1β, IL-6, TNF-α and INF-γ) in patients undergoing cardiac surgery for up to 24 h after CPB. In addition, randomized clinical studies have shown that in patients undergoing cardiac surgery with CPB, the intraoperative administration of DEX reduced the levels of pro-inflammatory cytokines during and after CPB, in addition to presenting possible renal and cardiac protection [108,109,110,111,112].

Some mechanisms are suggested for this effect, such as the inhibition of noradrenaline overflow and activation of the vagus nerve and the nicotinic acetylcholine receptor, which are related to the suppression of inflammatory cytokines [113,114]. In this sense, DEX is considered a promising candidate in modulating the inflammatory response, although more studies are needed to explore the effect of dexmedetomidine on the long-term prognosis of patients.

### 5.4. N-Acetylcysteine

N-acetylcysteine (NAC) is an acetyl derivative of L-cysteine with an active mercapto group. Although it is widely used as a mucolytic in respiratory syndromes, it has recently gained prominence, as studies have shown that the use of NAC prevents oxidative damage, inhibits apoptosis and the inflammatory response, and promotes glutathione synthesis in cells, one of the main endogenous antioxidants [115,116,117,118]. Regarding the heart, NAC can improve the systolic function of myocardial cells and cardiac function, in addition to protecting ventricular and vascular remodeling [115,119,120,121,122].

In addition, NAC has exhibited important effects by reducing the levels of lactate and nitrogenous slags in the blood 24 h after cardiac surgery with CPB, suggesting a beneficial effect on peripheral and renal tissue perfusion [123]. Furthermore, the prophylactic use of NAC attenuates the liver damage induced by cardiopulmonary bypass during cardiac surgery, in addition to reducing the incidence of acute kidney injury in this type of surgery [93,124,125,126]. However, more clinical studies are needed to standardize necessary doses and treatment times, as well as monitoring possible unwanted effects.

### 5.5. Nitric Oxide (NO)

NO has emerged as a promising alternative for protecting organs in cardiac surgeries, especially the kidneys and the heart itself. NO is formed in the human body from the oxidation of L-arginine via NO synthases, which are present in three isoforms (neuronal, endothelial and inducible) [127,128]. Administered via inhalation, it generates selective pulmonary vasodilation, whereas, due to its short half-life, it does not have systemic action. However, its metabolites can circulate throughout the body and act on more distant organs. Currently, inhaled NO is not recommended for routine use in patients under mechanical ventilation, but it is commonly used in pulmonary arterial hypertension crises and in persistent hypoxemia in acute respiratory distress syndrome (ARDS) [127].

For a long time, the possible deleterious effects of NO on cardiac muscle were marked, due to the formation of free radicals from its degradation. However, more robust studies have shown worsening of ischemia/reperfusion injuries when NO synthases are inhibited and improved outcomes with the administration of exogenous NO [127,129].

The clinical applicability of exogenous NO in the context of cardiac surgeries with PCB still has few studies, but with promising results. In randomized clinical trials of NO administration in patients undergoing cardiac surgery with CPB, the development of acute kidney injury was lower compared to the placebo groups [130,131]. In addition, recent studies have shown that the use of NO during CPB can contribute to cardioprotection, with significant reductions in troponin I levels and of the low cardiac output syndrome [132,133,134,135].

Thus, even with increasing evidence, there remain significant gaps, and more high-quality, multicenter, high-volume research is needed in both adult and pediatric populations.

### 5.6. Vitamin C

Ascorbic acid has a great ability to donate electrons, which makes it a potent antioxidant capable of interrupting cascades of free radicals that cause lipid peroxidation. It also contributes to the immune system in processes such as neutrophil chemotaxis, phagocytosis by lymphocytes and cell renewal [136,137]. In addition, vitamin C can phosphorylate the signaling pathway in erythrocytes, in addition to stimulating endothelial nitric oxide production, which can reduce blood loss and vasoplegic syndrome [138,139].

Furthermore, studies have been demonstrated the benefit of vitamin C supplementation, contributing to the reduction of arrhythmias (mainly atrial fibrillation) and duration of mechanical ventilation, ICU and hospital stay, despite not having demonstrated an improvement in mortality [138,139,140,141,142]. Although its use is promising, even with limitations, studies that assess the form of administration, whether in bolus or continuous infusion, before, during or after CPB are necessary to better assess the effectiveness of vitamin C supplementation.

### 5.7. Vitamin E

Vitamin E comes in different forms (isomers), with α-tocopherol having the highest antioxidant potential. These isomers are present in cell membranes and have an antioxidant action by inhibiting lipid peroxidation. It has already been demonstrated that serum levels of vitamin E are reduced during and after cardiac surgery; however, the primary outcomes linked to its supplementation are still conflicting despite good experimental results, mainly linked to α-tocopherol [143,144,145,146].

## 6. Conclusions

Cardiac surgery using cardiopulmonary bypass, although not perfect, remains essential within intraoperative management. The inflammatory state and the production of reactive oxygen species and oxidative stress remain challenges within the medical sciences. In addition, the use of pharmacological strategies with antioxidant potential that aim to reduce these radicals have a promising potential in reducing CPB complications such as the vasoplegic syndrome. Thus, more research needs to be carried out, whether in basic science or randomized controlled clinical studies, in addition to more rigid intraoperative management.

## Data Availability

No new data were created or analyzed in this study. Data sharing is not applicable to this article.

## References

[1] Hessel E.A. (2019). What’s New in Cardiopulmonary Bypass. J. Cardiothorac. Vasc. Anesth..

[2] Evora P.R.B., Bottura C., Arcêncio L., Albuquerque A.A.S., Évora P.M., Rodrigues A.J. (2016). Key Points for Curbing Cardiopulmonary Bypass Inflammation. Acta Cir. Bras..

[3] Borst C., Grundeman P.F. (1999). Minimally Invasive Coronary Artery Bypass Grafting. Circulation.

[4] Kowalik M.M., Lango R., Siondalski P., Chmara M., Brzeziński M., Lewandowski K., Jagielak D., Klapkowski A., Rogowski J. (2018). Clinical, Biochemical and Genetic Risk Factors for 30-Day and 5-Year Mortality in 518 Adult Patients Subjected to Cardiopulmonary Bypass during Cardiac Surgery—The INFLACOR Study. Acta Biochim. Pol..

[5] Long D., Jenkins E., Griffith K. (2015). Perfusionist Techniques of Reducing Acute Kidney Injury Following Cardiopulmonary Bypass: An Evidence-Based Review. Perfusion.

[6] Goodyear-Bruch C., Pierce J.D. (2002). Oxidative Stress in Critically Ill Patients. Am. J. Crit. Care.

[7] Karu I., Taal G., Zilmer K., Pruunsild C., Starkopf J., Zilmer M. (2010). Inflammatory/Oxidative Stress during the First Week after Different Types of Cardiac Surgery. Scand. Cardiovasc. J..

[8] Descamps-Latscha B., Drüeke T., Witko-Sarsat V. (2001). Dialysis-Induced Oxidative Stress: Biological Aspects, Clinical Consequences, and Therapy. Semin. Dial..

[9] Baehner T., Boehm O., Probst C., Poetzsch B., Hoeft A., Baumgarten G., Knuefermann P. (2012). Kardiopulmonaler Bypass in Der Herzchirurgie. Anaesthesist.

[10] Murphy G.J., Angelini G.D. (2004). Side Effects of Cardiopulmonary Bypass:. What Is the Reality?. J. Card. Surg..

[11] Busse L.W., Barker N., Petersen C. (2020). Vasoplegic Syndrome Following Cardiothoracic Surgery—Review of Pathophysiology and Update of Treatment Options. Crit. Care.

[12] Zakkar M., Guida G., Suleiman M.-S., Angelini G.D. (2015). Cardiopulmonary Bypass and Oxidative Stress. Oxidative Med. Cell. Longev..

[13] Gilbert M., Lema G. (2011). Vasoplegic Syndrome and Its Treatment with Vasopressin during Cardiac Surgery with Cardiopulmonary Bypass. Rev. Med. Chil..

[14] Rodrigues C.C.T.D.R. (2018). Systemic changes associated with cardiopulmonary bypass (CPB). Multidiscip. Core Sci. J. Knowl..

[15] Lima G., Cuervo M. (2019). Mecanismo Da Circulação Extracorpórea e Eventos Neurológicos Em Cirurgia Cardíaca. Rev. Soc. Port. Anestesiol..

[16] Martinez G., Whitbread J. (2012). Cardiopulmonary Bypass. Anaesth. Intensive Care Med..

[17] Çelik M., A Max S., Durko A.P., Mahtab E.A.F. (2021). Surgical Setup for Cardiopulmonary Bypass through Central Cannulation. Multimed. Man. Cardiothorac. Surg..

[18] Mota A.L., Rodrigues A.J., Évora P.R.B. (2008). Circulação Extracorpórea Em Adultos No Século XXI: Ciência, Arte Ou Empirismo?. Rev. Bras. Cir. Cardiovasc..

[19] Dekker N.A.M., van Leeuwen A.L.I., van de Ven P.M., de Vries R., Hordijk P.L., Boer C., van den Brom C.E. (2020). Pharmacological Interventions to Reduce Edema Following Cardiopulmonary Bypass: A Systematic Review and Meta-Analysis. J. Crit. Care.

[20] Barak M., Katz Y. (2005). Microbubbles. Chest.

[21] Biazzotto C.B., Brudniewski M., Schmidt A.P., Auler Júnior J.O.C. (2006). Hipotermia No Período Peri-Operatório. Rev. Bras. Anestesiol..

[22] Carneiro T.d.C. (2021). Hipotermia Na Circulação Extracorpórea Em Cirurgia Cardíaca. Res. Soc. Dev..

[23] da Costa Soares L.C., Ribas D., Spring R., Silva J.M.F.d., Miyague N.I. (2010). Perfil Clínico Da Resposta Inflamatória Sistêmica Após Cirurgia Cardíaca Pediátrica Com Circulação Extracorpórea. Arq. Bras. Cardiol..

[24] Pontes J.C.D.V., da Silva G.V.R., Benfatti R.A., Machado N.P., Pontelli R., Pontes E.R.J.C. (2007). Fatores de Risco No Desenvolvimento de Insuficiência Renal Aguda Após Cirurgia de Revascularização Miocárdica Com CEC. Rev. Bras. Cir. Cardiovasc..

[25] Santos F.O., Silveira M.A., Maia R.B., Monteiro M.D.C., Martinelli R. (2004). Insuficiência Renal Aguda Após Cirurgia de Revascularização Miocárdica Com Circulação Extracorpórea: Incidência, Fatores de Risco e Mortalidade. Arq. Bras. Cardiol..

[26] Andrade A.Y.T., de Lima Tanaka P.S., Poveda V.D.B., Turrini R.N.T. (2019). Complicações No Pós-Operatório Imediato de Revascularização Do Miocárdio. Rev. Sobecc.

[27] Torrati F.G., Dantas R.A.S. (2012). Circulação Extracorpórea e Complicações No Período Pós-Operatório Imediato de Cirurgias Cardíacas. Acta Paul. Enferm..

[28] Padovani C., Cavenaghi O.M. (2011). Recrutamento Alveolar Em Pacientes No Pós-Operatório Imediato de Cirurgia Cardíaca. Rev. Bras. Cir. Cardiovasc..

[29] Machado L.B., Negri E.M., Bonafé W.W., Santos L.M., Malbouisson L.M.S., Carmona M.J.C. (2011). Avaliação Dos Níveis de Citocinas e Da Função Pulmonar de Pacientes Submetidos à Cirurgia Cardíaca Com Circulação Extracorpórea. Rev. Bras. Anestesiol..

[30] Godinho A.S., Alves A.S., Pereira A.J., Pereira T.S. (2012). Cirurgia de Revascularização Miocárdica Com Circulação Extracorpórea versus Sem Circulação Extracorpórea: Uma Metanálise. Arq. Bras. Cardiol..

[31] Buggeskov K., Maltesen R., Rasmussen B., Hanifa M., Lund M., Wimmer R., Ravn H. (2018). Lung Protection Strategies during Cardiopulmonary Bypass Affect the Composition of Blood Electrolytes and Metabolites—A Randomized Controlled Trial. J. Clin. Med..

[32] Bilal M., Haseeb A., Khan M.H., Khetpal A., Saad M., Arshad M.H., Dar M.I., Hasan N., Rafiq R., Sherwani M. (2016). Assessment of Blood Glucose and Electrolytes during Cardiopulmonary Bypass in Diabetic and Non-Diabetic Patients of Pakistan. Glob. J. Health Sci..

[33] Shahidi M., Bakhshandeh H., Rahmani K., Afkhamzadeh A. (2019). Hypomagnesaemia and Other Electrolytes Imbalances in Open and Closed Pediatrics Cardiac Surgery. Pak. J. Med. Sci..

[34] Mustafa I., Roth H., Hanafiah A., Hakim T., Anwar M., Siregar E., Leverve X.M. (2003). Effect of Cardiopulmonary Bypass on Lactate Metabolism. Intensive Care Med..

[35] Stephens E.H., Epting C.L., Backer C.L., Wald E.L. (2020). Hyperlactatemia: An Update on Postoperative Lactate. World J. Pediatr. Congenit. Heart Surg..

[36] Demers P., Elkouri S., Martineau R., Couturier A., Cartier R. (2000). Outcome with High Blood Lactate Levels during Cardiopulmonary Bypass in Adult Cardiac Operation. Ann. Thorac. Surg..

[37] Clingan S., Reagor J., Lombardi J. (2019). Retrospective Analysis of Cardiac Index and Lactate Production on Cardiopulmonary Bypass for a Congenital Cardiac Patient Population. Perfusion.

[38] Adeva-Andany M., López-Ojén M., Funcasta-Calderón R., Ameneiros-Rodríguez E., Donapetry-García C., Vila-Altesor M., Rodríguez-Seijas J. (2014). Comprehensive Review on Lactate Metabolism in Human Health. Mitochondrion.

[39] Greco G., Kirkwood K.A., Gelijns A.C., Moskowitz A.J., Lam D.W. (2018). Diabetes Is Associated With Reduced Stress Hyperlactatemia in Cardiac Surgery. Diabetes Care.

[40] Giacinto O., Satriano U., Nenna A., Spadaccio C., Lusini M., Mastroianni C., Nappi F., Chello M. (2019). Inflammatory Response and Endothelial Dysfunction Following Cardiopulmonary Bypass: Pathophysiology and Pharmacological Targets. Recent Pat. Inflamm. Allergy Drug Discov..

[41] Becker A.C., Lantz C.W., Forbess J.M., Epting C.L., Thorp E.B. (2021). Cardiopulmonary Bypass–Induced Inflammation and Myocardial Ischemia and Reperfusion Injury Stimulates Accumulation of Soluble MER. Pediatr. Crit. Care Med..

[42] Millar J.E., Fanning J.P., McDonald C.I., McAuley D.F., Fraser J.F. (2016). The Inflammatory Response to Extracorporeal Membrane Oxygenation (ECMO): A Review of the Pathophysiology. Crit. Care.

[43] Phan S.H., Gannon D.E., Ward P.A., Karmiol S. (1992). Mechanism of Neutrophil-Induced Xanthine Dehydrogenase to Xanthine Oxidase Conversion in Endothelial Cells: Evidence of a Role for Elastase. Am. J. Respir. Cell Mol. Biol..

[44] Cyr A.R., Huckaby L.V., Shiva S.S., Zuckerbraun B.S. (2020). Nitric Oxide and Endothelial Dysfunction. Crit. Care Clin..

[45] Ayıkgöz Y., Salih Aydın M., Kankılıç N., Temiz E. (2021). Nuclear Factor Erythroid 2-Related Factor 2 (Nrf2), Tumor Necrosis Factor Alpha Protein (TNF-α), Heme Oxygenase-1 (HO-1) Gene Expressions during Cardiopulmonary Bypass. Gene.

[46] Doyle A.J., Hunt B.J. (2018). Current Understanding of How Extracorporeal Membrane Oxygenators Activate Haemostasis and Other Blood Components. Front. Med..

[47] Zimmerman G.A., McIntyre T.M., Prescott S.M. (1985). Thrombin Stimulates the Adherence of Neutrophils to Human Endothelial Cells in Vitro. J. Clin. Investig.

[48] Kaplanski G., Fabrigoule M., Boulay V., Dinarello C.A., Bongrand P., Kaplanski S., Farnarier C. (1997). Thrombin Induces Endothelial Type II Activation in Vitro: IL-1 and TNF-Alpha-Independent IL-8 Secretion and E-Selectin Expression. J. Immunol..

[49] Prescott S.M., Zimmerman G.A., McIntyre T.M. (1984). Human Endothelial Cells in Culture Produce Platelet-Activating Factor (1-Alkyl-2-Acetyl-Sn-Glycero-3-Phosphocholine) When Stimulated with Thrombin. Proc. Natl. Acad. Sci. USA.

[50] Esper S.A., Subramaniam K., Tanaka K.A. (2014). Pathophysiology of Cardiopulmonary Bypass. Semin. Cardiothorac. Vasc. Anesth..

[51] Hatami S., Hefler J., Freed D.H. (2022). Inflammation and Oxidative Stress in the Context of Extracorporeal Cardiac and Pulmonary Support. Front. Immunol..

[52] Ng C.S., Wan S. (2012). Limiting Inflammatory Response to Cardiopulmonary Bypass: Pharmaceutical Strategies. Curr. Opin. Pharmacol..

[53] Kawahito K., Kobayashi E., Ohmori M., Harada K., Kitoh Y., Fujimura A., Fuse K. (2000). Enhanced Responsiveness of Circulatory Neutrophils After Cardiopulmonary Bypass: Increased Aggregability and Superoxide Producing Capacity. Artif. Organs.

[54] Li J.-M., Mullen A.M., Yun S., Wientjes F., Brouns G.Y., Thrasher A.J., Shah A.M. (2002). Essential Role of the NADPH Oxidase Subunit P47 Phox in Endothelial Cell Superoxide Production in Response to Phorbol Ester and Tumor Necrosis Factor-α. Circ. Res..

[55] Suleiman M.-S., Zacharowski K., Angelini G.D. (2008). Inflammatory Response and Cardioprotection during Open-Heart Surgery: The Importance of Anaesthetics. Br. J. Pharmacol..

[56] Baskurt O.K., Meiselman H.J. (2003). Blood Rheology and Hemodynamics. Semin. Thromb. Hemost..

[57] Morariu A., Gu Y., Huet R., Siemons W., Rakhorst G., Oeveren W. (2004). Red Blood Cell Aggregation during Cardiopulmonary Bypass: A Pathogenic Cofactor in Endothelial Cell Activation?. Eur. J. Cardio-Thorac. Surg..

[58] Byrne J. (2004). Risk Factors and Outcomes for “vasoplegia Syndrome” Following Cardiac Transplantation. Eur. J. Cardio-Thorac. Surg..

[59] Levin M.A., Lin H.-M., Castillo J.G., Adams D.H., Reich D.L., Fischer G.W. (2009). Early On–Cardiopulmonary Bypass Hypotension and Other Factors Associated With Vasoplegic Syndrome. Circulation.

[60] Omar S., Zedan A., Nugent K. (2015). Cardiac Vasoplegia Syndrome: Pathophysiology, Risk Factors and Treatment. Am. J. Med. Sci..

[61] Shaefi S., Mittel A., Klick J., Evans A., Ivascu N.S., Gutsche J., Augoustides J.G.T. (2018). Vasoplegia After Cardiovascular Procedures—Pathophysiology and Targeted Therapy. J. Cardiothorac. Vasc. Anesth..

[62] Landry D.W., Oliver J.A. (2001). The Pathogenesis of Vasodilatory Shock. N. Engl. J. Med..

[63] Vincent J.-L., De Backer D. (2013). Circulatory Shock. N. Engl. J. Med..

[64] Weis F., Kilger E., Beiras-Fernandez A., Nassau K., Reuter D., Goetz A., Lamm P., Reindl L., Briegel J. (2006). Association between Vasopressor Dependence and Early Outcome in Patients after Cardiac Surgery. Anaesthesia.

[65] Leyh R.G., Kofidis T., Strüber M., Fischer S., Knobloch K., Wachsmann B., Hagl C., Simon A.R., Haverich A. (2003). Methylene Blue: The Drug of Choice for Catecholamine-Refractory Vasoplegia after Cardiopulmonary Bypass. J. Thorac. Cardiovasc. Surg..

[66] Levy B., Fritz C., Tahon E., Jacquot A., Auchet T., Kimmoun A. (2018). Vasoplegia Treatments: The Past, the Present, and the Future. Crit. Care.

[67] Elenkov I.J., Wilder R.L., Chrousos G.P., Vizi E.S. (2000). The Sympathetic Nerve--an Integrative Interface between Two Supersystems: The Brain and the Immune System. Pharmacol. Rev..

[68] Liu J., Hughes T.E., Sessa W.C. (1997). The First 35 Amino Acids and Fatty Acylation Sites Determine the Molecular Targeting of Endothelial Nitric Oxide Synthase into the Golgi Region of Cells: A Green Fluorescent Protein Study. J. Cell Biol..

[69] Spink J., Cohen J., Evans T.J. (1995). The Cytokine Responsive Vascular Smooth Muscle Cell Enhancer of Inducible Nitric Oxide Synthase. J. Biol. Chem..

[70] Datt V., Wadhhwa R., Sharma V., Virmani S., Minhas H.S., Malik S. (2021). Vasoplegic Syndrome after Cardiovascular Surgery: A Review of Pathophysiology and Outcome-oriented Therapeutic Management. J. Card. Surg..

[71] Anavi S., Tirosh O. (2020). INOS as a Metabolic Enzyme under Stress Conditions. Free Radic. Biol. Med..

[72] Green S.J., Scheller L.F., Marletta M.A., Seguin M.C., Klotz F.W., Slayter M., Nelson B.J., Nacy C.A. (1994). Nitric Oxide: Cytokine-Regulation of Nitric Oxide in Host Resistance to Intracellular Pathogens. Immunol. Lett..

[73] Forstermann U., Sessa W.C. (2012). Nitric Oxide Synthases: Regulation and Function. Eur. Heart J..

[74] Ko E.A., Han J., Jung I.D., Park W.S. (2008). Physiological Roles of K+ Channels in Vascular Smooth Muscle Cells. J. Smooth Muscle Res..

[75] GAO M., XIE B., GU C., LI H., ZHANG F., YU Y. (2015). Targeting the Proinflammatory Cytokine Tumor Necrosis Factor-α to Alleviate Cardiopulmonary Bypass-Induced Lung Injury (Review). Mol. Med. Rep..

[76] Haeffner-Cavaillon N., Roussellier N., Ponzio O., Carreno M.P., Laude M., Carpentier A., Kazatchkine M.D. (1989). Induction of Interleukin-1 Production in Patients Undergoing Cardiopulmonary Bypass. J. Thorac. Cardiovasc. Surg..

[77] Hill G.E., Whitten C.W., Landers D.F. (1997). The Influence of Cardiopulmonary Bypass on Cytokines and Cell-Cell Communication. J. Cardiothorac. Vasc. Anesth..

[78] Liu X., Yang L., Wang L., Guo Q. (2021). RETRACTED: Oleocanthal Protects against Neuronal Inflammation and Cardiopulmonary Bypass Surgery-Induced Brain Injury in Rats by Regulating the NLRP3 Pathway. Restor. Neurol. Neurosci..

[79] Pacher P., Beckman J.S., Liaudet L. (2007). Nitric Oxide and Peroxynitrite in Health and Disease. Physiol. Rev..

[80] Kerbaul F., Guidon C., Lejeune P.J., Mollo M., Mesana T., Gouin F. (2002). Hyperprocalcitonemia Is Related to Noninfectious Postoperative Severe Systemic Inflammatory Response Syndrome Associated with Cardiovascular Dysfunction after Coronary Artery Bypass Graft Surgery. J. Cardiothorac. Vasc. Anesth..

[81] Ma H., Dong Y., Sun K., Wang S., Zhang Z. (2022). Protective Effect of MiR-146 on Renal Injury Following Cardiopulmonary Bypass in Rats through Mediating NF-ΚB Signaling Pathway. Bioengineered.

[82] Ammar M.A., Ammar A.A., Wieruszewski P.M., Bissell B.D., Long M.T., Albert L., Khanna A.K., Sacha G.L. (2022). Timing of Vasoactive Agents and Corticosteroid Initiation in Septic Shock. Ann. Intensive Care.

[83] Papazisi O., Palmen M., Danser A.H.J. (2022). The Use of Angiotensin II for the Treatment of Post-Cardiopulmonary Bypass Vasoplegia. Cardiovasc. Drugs Ther..

[84] Jochberger S., Velik-Salchner C., Mayr V.D., Luckner G., Wenzel V., Falkensammer G., Ulmer H., Morgenthaler N., Hasibeder W., Dünser M.W. (2009). The Vasopressin and Copeptin Response in Patients with Vasodilatory Shock after Cardiac Surgery: A Prospective, Controlled Study. Intensive Care Med..

[85] Colson P.H., Bernard C., Struck J., Morgenthaler N.G., Albat B., Guillon G. (2011). Post Cardiac Surgery Vasoplegia Is Associated with High Preoperative Copeptin Plasma Concentration. Crit. Care.

[86] Landry D.W., Levin H.R., Gallant E.M., Ashton R.C., Seo S., D’Alessandro D., Oz M.C., Oliver J.A. (1997). Vasopressin Deficiency Contributes to the Vasodilation of Septic Shock. Circulation.

[87] Davies N.W. (1990). Modulation of ATP-Sensitive K+ Channels in Skeletal Muscle by Intracellular Protons. Nature.

[88] Ohkawa F., Ikeda U., Kanbe T., Kawasaki K., Shimada K. (1995). Effects of Inflammatory Cytokines on Vascular Tone. Cardiovasc. Res..

[89] Kilger E., Weis F., Briegel J., Frey L., Goetz A.E., Reuter D., Nagy A., Schuetz A., Lamm P., Knoll A. (2003). Stress Doses of Hydrocortisone Reduce Severe Systemic Inflammatory Response Syndrome and Improve Early Outcome in a Risk Group of Patients after Cardiac Surgery. Crit. Care Med..

[90] Träger K., Fritzler D., Fischer G., Schröder J., Skrabal C., Liebold A., Reinelt H. (2016). Treatment of Post-Cardiopulmonary Bypass SIRS by Hemoadsorption: A Case Series. Int. J. Artif. Organs.

[91] Manzanares W., Dhaliwal R., Jiang X., Murch L., Heyland D.K. (2012). Antioxidant Micronutrients in the Critically Ill: A Systematic Review and Meta-Analysis. Crit. Care.

[92] Visser J., Labadarios D., Blaauw R. (2011). Micronutrient Supplementation for Critically Ill Adults: A Systematic Review and Meta-Analysis. Nutrition.

[93] Kumar R., Bansal M., Nath S.S., Kumar V., Malviya D., Srivastava D. (2021). N-Acetylcysteine Supplementation for the Prevention of Postoperative Liver Dysfunction after On-Pump Cardiac Surgery. Turk. J. Anaesthesiol. Reanim..

[94] McDonald C.I., Fraser J.F., Coombes J.S., Fung Y.L. (2014). Oxidative Stress during Extracorporeal Circulation. Eur. J. Cardio-Thorac. Surg..

[95] Türker F.S., Doğan A., Ozan G., Kıbar K., Erışır M. (2016). Change in Free Radical and Antioxidant Enzyme Levels in the Patients Undergoing Open Heart Surgery with Cardiopulmonary Bypass. Oxidative Med. Cell. Longev..

[96] Alsatli R. (2012). Mini Cardiopulmonary Bypass: Anesthetic Considerations. Anesth. Essays Res..

[97] Mueller X. (2002). A New Concept of Integrated Cardiopulmonary Bypass Circuit. Eur. J. Cardio-Thorac. Surg..

[98] Anastasiadis K., Murkin J., Antonitsis P., Bauer A., Ranucci M., Gygax E., Schaarschmidt J., Fromes Y., Philipp A., Eberle B. (2016). Use of Minimal Invasive Extracorporeal Circulation in Cardiac Surgery: Principles, Definitions and Potential Benefits. A Position Paper from the Minimal Invasive Extra-Corporeal Technologies International Society (MiECTiS). Interact. Cardiovasc. Thorac. Surg..

[99] Cheng T., Barve R., Cheng Y.W.M., Ravendren A., Ahmed A., Toh S., Goulden C.J., Harky A. (2021). Conventional versus Miniaturized Cardiopulmonary Bypass: A Systematic Review and Meta-Analysis. JTCVS Open.

[100] Walski T., Drohomirecka A., Bujok J., Czerski A., Wąż G., Trochanowska-Pauk N., Gorczykowski M., Cichoń R., Komorowska M. (2018). Low-Level Light Therapy Protects Red Blood Cells Against Oxidative Stress and Hemolysis During Extracorporeal Circulation. Front. Physiol..

[101] Drohomirecka A., Iwaszko A., Walski T., Pliszczak-Król A., Wąż G., Graczyk S., Gałecka K., Czerski A., Bujok J., Komorowska M. (2018). Low-Level Light Therapy Reduces Platelet Destruction during Extracorporeal Circulation. Sci. Rep..

[102] Chludzińska L., Ananicz E., Jarosawska A., Komorowska M. (2005). Near-Infrared Radiation Protects the Red Cell Membrane against Oxidation. Blood Cells Mol. Dis..

[103] Walski T., Dyrda A., Dzik M., Chludzińska L., Tomków T., Mehl J., Detyna J., Gałecka K., Witkiewicz W., Komorowska M. (2015). Near Infrared Light Induces Post-Translational Modifications of Human Red Blood Cell Proteins. Photochem. Photobiol. Sci..

[104] Hall R. (2013). Identification of Inflammatory Mediators and Their Modulation by Strategies for the Management of the Systemic Inflammatory Response During Cardiac Surgery. J. Cardiothorac. Vasc. Anesth..

[105] Li P., Han J., Zhang D., Cao S., Su C. (2019). Effects of Dexmedetomidine on Oxidative Stress and Inflammatory Response in Lungs during Mechanical Ventilation in COPD Rats. Exp. Ther. Med..

[106] CAN M., GUL S., BEKTAS S., HANCI V., ACIKGOZ S. (2009). Effects of Dexmedetomidine or Methylprednisolone on Inflammatory Responses in Spinal Cord Injury. Acta Anaesthesiol. Scand..

[107] Bulow N.M.H., Colpo E., Pereira R.P., Correa E.F.M., Waczuk E.P., Duarte M.F., Rocha J.B.T. (2016). Dexmedetomidine Decreases the Inflammatory Response to Myocardial Surgery under Mini-Cardiopulmonary Bypass. Braz. J. Med. Biol. Res..

[108] Ueki M., Kawasaki T., Habe K., Hamada K., Kawasaki C., Sata T. (2014). The Effects of Dexmedetomidine on Inflammatory Mediators after Cardiopulmonary Bypass. Anaesthesia.

[109] Kim S., Park S.J., Nam S.B., Song S.-W., Han Y., Ko S., Song Y. (2021). Pulmonary Effects of Dexmedetomidine Infusion in Thoracic Aortic Surgery under Hypothermic Circulatory Arrest: A Randomized Placebo-Controlled Trial. Sci. Rep..

[110] Zhai M., Kang F., Han M., Huang X., Li J. (2017). The Effect of Dexmedetomidine on Renal Function in Patients Undergoing Cardiac Valve Replacement under Cardiopulmonary Bypass: A Double-Blind Randomized Controlled Trial. J. Clin. Anesth..

[111] Turan A., Duncan A., Leung S., Karimi N., Fang J., Mao G., Hargrave J., Gillinov M., Trombetta C., Ayad S. (2020). Dexmedetomidine for Reduction of Atrial Fibrillation and Delirium after Cardiac Surgery (DECADE): A Randomised Placebo-Controlled Trial. Lancet.

[112] Peng K., Shen Y., Ying Y., Kiaii B., Rodriguez V., Boyd D., Applegate R.L., Lubarsky D.A., Zhang Z., Xia Z. (2021). Perioperative Dexmedetomidine and 5-Year Survival in Patients Undergoing Cardiac Surgery. Br. J. Anaesth..

[113] Moura E., Afonso J., Hein L., Vieira-Coelho M.A. (2006). α 2 -Adrenoceptor Subtypes Involved in the Regulation of Catecholamine Release from the Adrenal Medulla of Mice. Br. J. Pharmacol..

[114] Xiang H., Hu B., Li Z., Li J. (2014). Dexmedetomidine Controls Systemic Cytokine Levels through the Cholinergic Anti-Inflammatory Pathway. Inflammation.

[115] Cai Z., Shi T., Zhuang R., Fang H., Jiang X., Shao Y., Zhou H. (2018). Protective Effect of N-Acetylcysteine Activated Carbon Release Microcapsule on Myocardial Ischemia-Reperfusion Injury in Rats. Exp. Ther. Med..

[116] Blasi F., Page C., Rossolini G.M., Pallecchi L., Matera M.G., Rogliani P., Cazzola M. (2016). The Effect of N -Acetylcysteine on Biofilms: Implications for the Treatment of Respiratory Tract Infections. Respir. Med..

[117] Paintlia M.K., Paintlia A.S., Contreras M.A., Singh I., Singh A.K. (2008). Lipopolysaccharide-Induced Peroxisomal Dysfunction Exacerbates Cerebral White Matter Injury: Attenuation by N-Acetyl Cysteine. Exp. Neurol..

[118] Samuni Y., Goldstein S., Dean O.M., Berk M. (2013). The Chemistry and Biological Activities of N-Acetylcysteine. Biochim. Et Biophys. Acta (BBA)-Gen. Subj..

[119] Yi X., Cui X., Wu P., Wang S., Wang G., Yang X., Yang F., Zheng S., Li Z. (2013). Effects of N-Acetylcysteine on Apoptosis Induced by Myocardial Ischemia Reperfusion Injury in Rats’ Heart Transplantation. Chin. J. Reparative Reconstr. Surg..

[120] WU X.-Y., LUO A.-Y., ZHOU Y.-R., REN J.-H. (2014). N-Acetylcysteine Reduces Oxidative Stress, Nuclear Factor-ΚB Activity and Cardiomyocyte Apoptosis in Heart Failure. Mol. Med. Rep..

[121] Orhan G., Yapici N., Yuksel M., Sargin M., Şenay Ş., Yalçin A.S., Aykaç Z., Aka S.A. (2006). Effects of N-Acetylcysteine on Myocardial Ischemia–Reperfusion Injury in Bypass Surgery. Heart Vessels.

[122] Giam B., Chu P.-Y., Kuruppu S., Smith A.I., Horlock D., Kiriazis H., Du X.-J., Kaye D.M., Rajapakse N.W. (2016). N- Acetylcysteine Attenuates the Development of Cardiac Fibrosis and Remodeling in a Mouse Model of Heart Failure. Physiol. Rep..

[123] Onk D., Özçelik F., Onk O.A., Günay M., Akarsu Ayazoğlu T., Ünver E. (2018). Assessment of Renal and Hepatic Tissue-Protective Effects of N-Acetylcysteine via Ammonia Metabolism: A Prospective Randomized Study. Med. Sci. Monit..

[124] Permeisari D. (2022). Future Insights of Pharmacological Prevention for AKI Post Cardiopulmonary Bypass Surgery (Based on PK/PD Approach). Front. Pharmacol..

[125] Tan S.I., Brewster D.J., Horrigan D., Sarode V. (2019). Pharmacological and Non-surgical Renal Protective Strategies for Cardiac Surgery Patients Undergoing Cardiopulmonary Bypass: A Systematic Review. ANZ J. Surg..

[126] Savluk O.F., Guzelmeric F., Yavuz Y., Cevirme D., Gurcu E., Ogus H., Orki T., Kocak T. (2017). N-Acetylcysteine versus Dopamine to Prevent Acute Kidney Injury after Cardiac Surgery in Patients with Preexisting Moderate Renal Insufficiency. Braz. J. Cardiovasc. Surg..

[127] Redaelli S., Magliocca A., Malhotra R., Ristagno G., Citerio G., Bellani G., Berra L., Rezoagli E. (2022). Nitric Oxide: Clinical Applications in Critically Ill Patients. Nitric Oxide.

[128] Kamenshchikov N.O., Duong N., Berra L. (2023). Nitric Oxide in Cardiac Surgery: A Review Article. Biomedicines.

[129] Bolli R. (2000). The Late Phase of Preconditioning. Circ. Res..

[130] Kamenshchikov N.O., Anfinogenova Y.J., Kozlov B.N., Svirko Y.S., Pekarskiy S.E., Evtushenko V.V., Lugovsky V.A., Shipulin V.M., Lomivorotov V.V., Podoksenov Y.K. (2022). Nitric Oxide Delivery during Cardiopulmonary Bypass Reduces Acute Kidney Injury: A Randomized Trial. J. Thorac. Cardiovasc. Surg..

[131] Loughlin J.M., Browne L., Hinchion J. (2022). The Impact of Exogenous Nitric Oxide during Cardiopulmonary Bypass for Cardiac Surgery. Perfusion.

[132] Hornik C.P. (2020). Invited Commentary: Efficacy of Nitric Oxide Administration During Neonatal Cardiopulmonary Bypass. World J. Pediatr. Congenit. Heart Surg..

[133] Checchia P.A., Bronicki R.A., Muenzer J.T., Dixon D., Raithel S., Gandhi S.K., Huddleston C.B. (2013). Nitric Oxide Delivery during Cardiopulmonary Bypass Reduces Postoperative Morbidity in Children—A Randomized Trial. J. Thorac. Cardiovasc. Surg..

[134] James C., Millar J., Horton S., Brizard C., Molesworth C., Butt W. (2016). Nitric Oxide Administration during Paediatric Cardiopulmonary Bypass: A Randomised Controlled Trial. Intensive Care Med..

[135] Kamenshchikov N.O., Mandel I.A., Podoksenov Y.K., Svirko Y.S., Lomivorotov V.V., Mikheev S.L., Kozlov B.N., Shipulin V.M., Nenakhova A.A., Anfinogenova Y.J. (2019). Nitric Oxide Provides Myocardial Protection When Added to the Cardiopulmonary Bypass Circuit during Cardiac Surgery: Randomized Trial. J. Thorac. Cardiovasc. Surg..

[136] Carr A., Maggini S. (2017). Vitamin C and Immune Function. Nutrients.

[137] Doseděl M., Jirkovský E., Macáková K., Krčmová L., Javorská L., Pourová J., Mercolini L., Remião F., Nováková L., Mladěnka P. (2021). Vitamin C—Sources, Physiological Role, Kinetics, Deficiency, Use, Toxicity, and Determination. Nutrients.

[138] Tai Y.-H., Wu H.-L., Chu Y.-H., Huang C.-H., Ho S.-T., Lin T.-C., Lu C.-C. (2022). Vitamin C Supplementation Attenuates Oxidative Stress and Improves Erythrocyte Deformability in Cardiac Surgery with Cardiopulmonary Bypass. Chin. J. Physiol..

[139] Wieruszewski P.M., Nei S.D., Maltais S., Schaff H.V., Wittwer E.D. (2018). Vitamin C for Vasoplegia After Cardiopulmonary Bypass: A Case Series. A A Pract..

[140] Ghorbaninezhad K., Bakhsha F., Yousefi Z., Halakou S., Mehrbakhsh Z. (2019). Comparison Effect of Tranexamic Acid (TA) and Tranexamic Acid Combined with Vitamin C (TXC) on Drainage Volume and Atrial Fibrillation Arrhythmia in Patients Undergoing Cardiac Bypass Surgery: Randomized Clinical Trial. Anesthesiol. Pain Med..

[141] Yanase F., Bitker L., Hessels L., Osawa E., Naorungroj T., Cutuli S.L., Young P.J., Ritzema J., Hill G., Latimer-Bell C. (2020). A Pilot, Double-Blind, Randomized, Controlled Trial of High-Dose Intravenous Vitamin C for Vasoplegia After Cardiac Surgery. J. Cardiothorac. Vasc. Anesth..

[142] Das D., Sen C., Goswami A. (2016). Effect of Vitamin C on Adrenal Suppression by Etomidate Induction in Patients Undergoing Cardiac Surgery: A Randomized Controlled Trial. Ann. Card. Anaesth..

[143] Miyazawa T., Burdeos G.C., Itaya M., Nakagawa K., Miyazawa T. (2019). Vitamin E: Regulatory Redox Interactions. IUBMB Life.

[144] Hill A., Borgs C., Fitzner C., Stoppe C. (2019). Perioperative Vitamin C and E Levels in Cardiac Surgery Patients and Their Clinical Significance. Nutrients.

[145] Hu X.-X., Fu L., Li Y., Lin Z.-B., Liu X., Wang J.-F., Chen Y.-X., Wang Z.-P., Zhang X., Ou Z.-J. (2015). The Cardioprotective Effect of Vitamin E (Alpha-Tocopherol) Is Strongly Related to Age and Gender in Mice. PLoS ONE.

[146] Zakkar M., Ascione R., James A.F., Angelini G.D., Suleiman M.S. (2015). Inflammation, Oxidative Stress and Postoperative Atrial Fibrillation in Cardiac Surgery. Pharmacol. Ther..

